# A personalized channel recommendation and scheduling system considering both section video clips and full video clips

**DOI:** 10.1371/journal.pone.0199748

**Published:** 2018-07-06

**Authors:** SeungGwan Lee, DaeHo Lee

**Affiliations:** Humanitas College, Kyung Hee University, Deogyeong-daero, Giheung-gu, Yongin-si, Gyeonggi-do, South Korea; Education University of Hong Kong, CHINA

## Abstract

With the convergence of various broadcasting systems, the amount of content available in mobile terminals including IPTV has significantly increased. In this paper, we propose a system that enables users to schedule programs considering both section video clips and full video clips based on the user detection method with similar preference. And, since the system constituting the contents can be classified according to the program, the proposed method can store a program desired by the user, and thus create and schedule a kind of individual channel. Experimental results show that the proposed method has a higher prediction accuracy; this is accomplished by comparing existing channel recommendation methods with the program recommendation methods proposed in this paper.

## Introduction

With the convergence of broadcasting and communication, the number of broadcasting programs that can be consumed in user TV terminals has significantly increased. In addition, through the provision of various contents such as live TV channels, IPTV services, or TV portals, the amount of program content available to users on TV terminals has greatly increased. One way for users to watch their favorite broadcast program contents is to search for program contents or through automatic recommendation [1]. In the case of content search, the user has to search for a keyword of the program, input it into the search engine, and wait for confirmation of the search result. Also, it is necessary to select a desired program from among the program contents presented in the search results. However, when using the automatic recommendation method, it is possible to reduce the amount of steps. When the recommendation is performed using business filtering, there is an advantage in that the user can present preferred program contents even if the user does not recognize it. This is possible because collaborative filtering performs recommendations using the preference program content of a similar preference user group.

In this paper, we propose an automatic recommendation algorithm based on collaborative filtering. The proposed system consists of offline and online calculation processes.

The proposed system understands the user’s tendencies considering the characteristics of real-time and VOD videos of the IPTV, recommends the optimal program to users with similar tendencies, and enables unique program scheduling based on this. Also, it is possible to recommend videos to the user considering both section video clips and full video clips.

This paper is structured as follows. In Chapter II, we first examine the content filtering approach and the collaborative filtering approach, which have both been previously studied. In Section III, we propose a new TV program recommendation algorithm. Section IV describes the experimental results and analysis. Section V presents the conclusions of this paper.

## Related works

In this section, we describe the existing research related to this paper. Section A explains the content-based approach and Section B explains the collaborative filtering technique.

### A. Content-based approach

The content-based approach is a system based on information retrieval technology. It analyzes the similarity between items and user preference by directly analyzing item content and recommending items to the customer based on this analysis [2–6].

**Fig 1:**
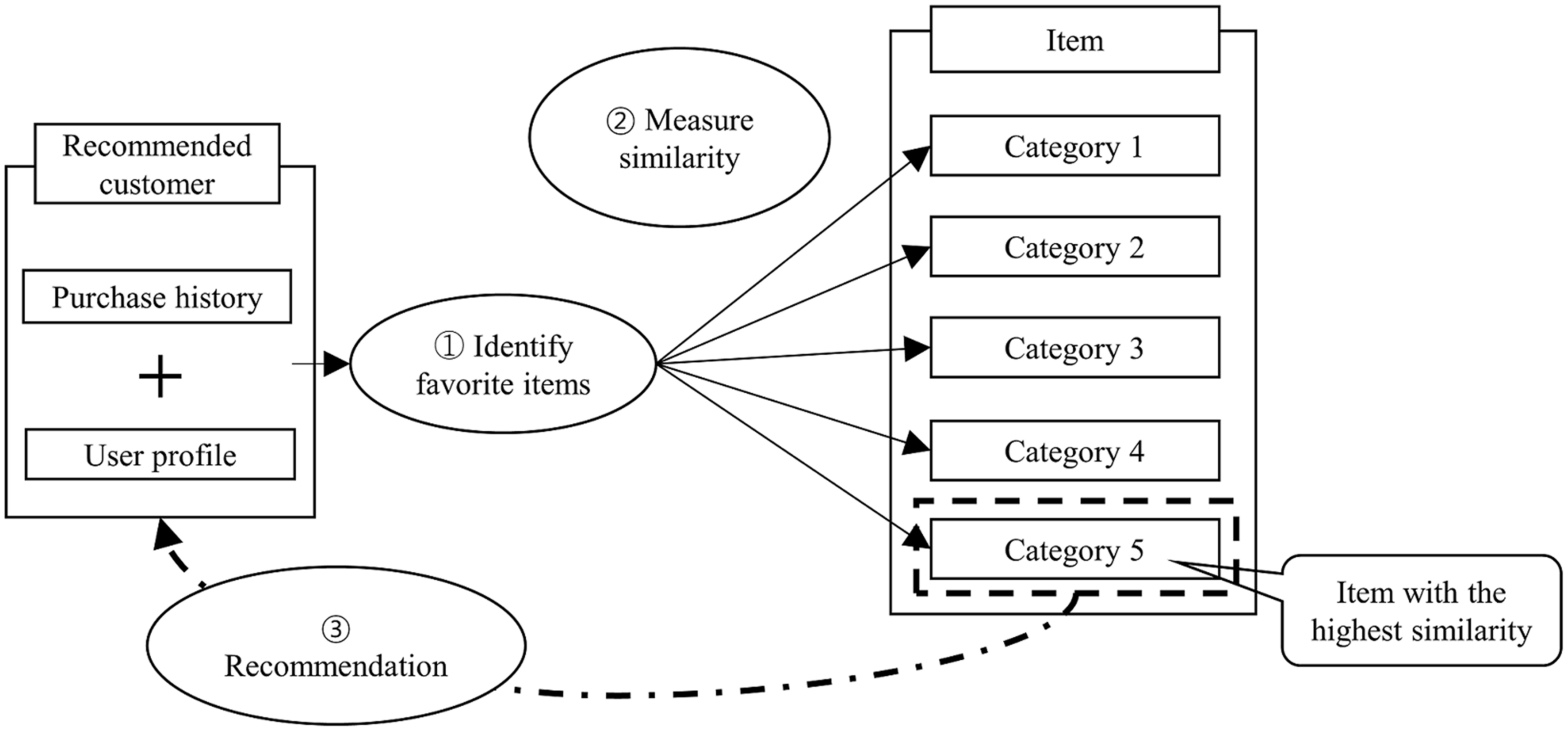
Content-based approach algorithm.

Fig 1 shows a recommendation scheme for a content-based approach [2]. A preference item is identified through the profile information input by the user, the user’s score of the item or the information generated based on the past purchase history, and then the item category is classified by the pre-selected criteria and the user’s preference item. Finally, items with the highest similarity are recommended (ex: Category 5 in Fig 1).

In the content-based approach, the past purchase history or profile information of the target is used to determine the preference of the recommendation target. In this case, since such an approach requires only the independent information of the customer, it is a recommendation technique that can be useful even when the information for other users is insufficient. However, if the preference through past purchasing history is required, there must be a sufficient number of item evaluation scores to create the recommendation. If the past purchase history is insufficient, the performance of the recommendation cannot be guaranteed.

If the purchase history and the profile information do not exist, the recommendation system of the content-based approach cannot be implemented [7, 8]. In addition, since the content-based approach finds similar products based on past purchasing histories of the customers to be recommended, it does not reflect the taste or preference of other users, and only the products that are similar to the purchased goods are recommended. Since there is no opportunity to access the new item attribute, diversity of the recommendation items cannot be guaranteed; this problem is known as over specialization [9]. In order to solve this problem, researches proposed an online hybrid recommender strategy with dynamic fuzzy clustering based on content boosted collaborative filtering algorithm [10], and proposed a novel probabilistic method for recommending items in the neighborhood-based collaborative filtering framework [11].

### B. Collaborative filtering

Collaborative filtering is one of the most commonly used recommendation systems [12–14]. It has been used in various contexts, such as Amazon.co.uk, Internet bookstores, MovieFinder, CDNOW.com, and internet movie recommendation sites, initially starting with Group-Lens from the University of Minnesota.

Collaborative filtering is a method of predicting the preference based on the similarity between users or items, specifically the premise that ‘customers with similar preferences for a particular item will show similar preferences for other items.’ While the content-based approach predicts estimates depending only on user and item information, the most important difference of collaborative filtering is that user preference is predicted using item evaluation information [15]. It is possible to guarantee diversity in recommended items because users who are similar in taste to other customers who are referred to are selected and their favorite items are recommended to the target customers.

**Fig 2:**
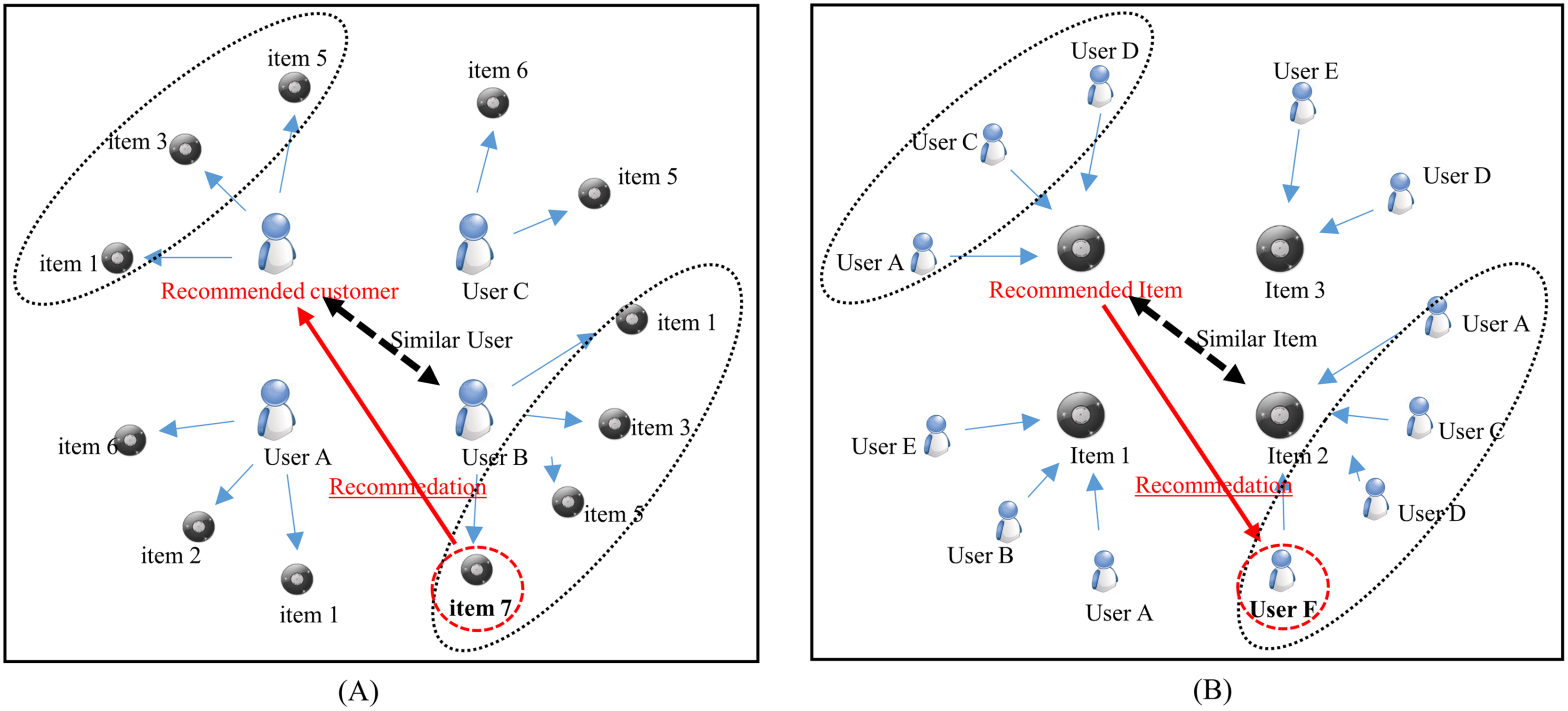
Collaborative filtering algorithm. (A) User-based collaborative filtering. (B) Item-based collaborative filtering.

Fig 2A shows the basic concept of user-based collaborative filtering. When the recommended target customer (i.e., the customer who is need of recommended items) is selected, the degree of similarity between the recommended customer and the other users is measured based on their purchase histories. In this process, the more similar items that are purchased, the higher the degree of similarity, and when this degree of similarity is measured for all users, the user with the highest similarity is selected as the neighbor. For example, in the case of Fig 2A, the user whose taste is closest to the recommendation target is ‘User B’ who purchased ‘Item 1’, ‘Item 3’ and ‘Item 5’. In the final stage of collaborative filtering, ‘Item 7’ is selected as a recommendation object, as ‘Item 7’ was purchased by ‘User B’ but not purchased by the recommendation target customer; this is accomplished by using the similarity measure as an item recommendation step.

Fig 2B shows the basic concept of item-based collaborative filtering. After selecting a similar item based on the recommendation target item, the recommendation target item is finally recommended to ‘User F’ who purchased the selected ‘Item 2’ but did not purchase the recommended item.

Collaborative filtering can be divided into memory-based collaborative filtering and model-based collaborative filtering [16, 17]. Memory-based collaborative filtering is a method of calculating the similarity between users in the manner described above and recommending items selected by users with high similarity. Model-based collaborative filtering is based on memory-based collaborative filtering, but utilizes machine learning or data mining techniques at the level of clustering, classification, and prediction. In order to overcome the various problems of applying the memory-based collaborative filtering, the proposed model is Bayesian and utilizes both linear regression analysis and a Markov decision process based on probability theory and statistics [18, 19].

In this chapter, we first describe the features of collaborative filtering, and then describe memory-based collaborative filtering and model-based collaborative filtering.

#### • Features of collaborative filtering

The most commonly used method in the Collaborative Filtering (CF) method is Matrix Factorization (MF) [20, 21]. In previous papers, researchers use MF to solve data dimensionality.

**Fig 3:**
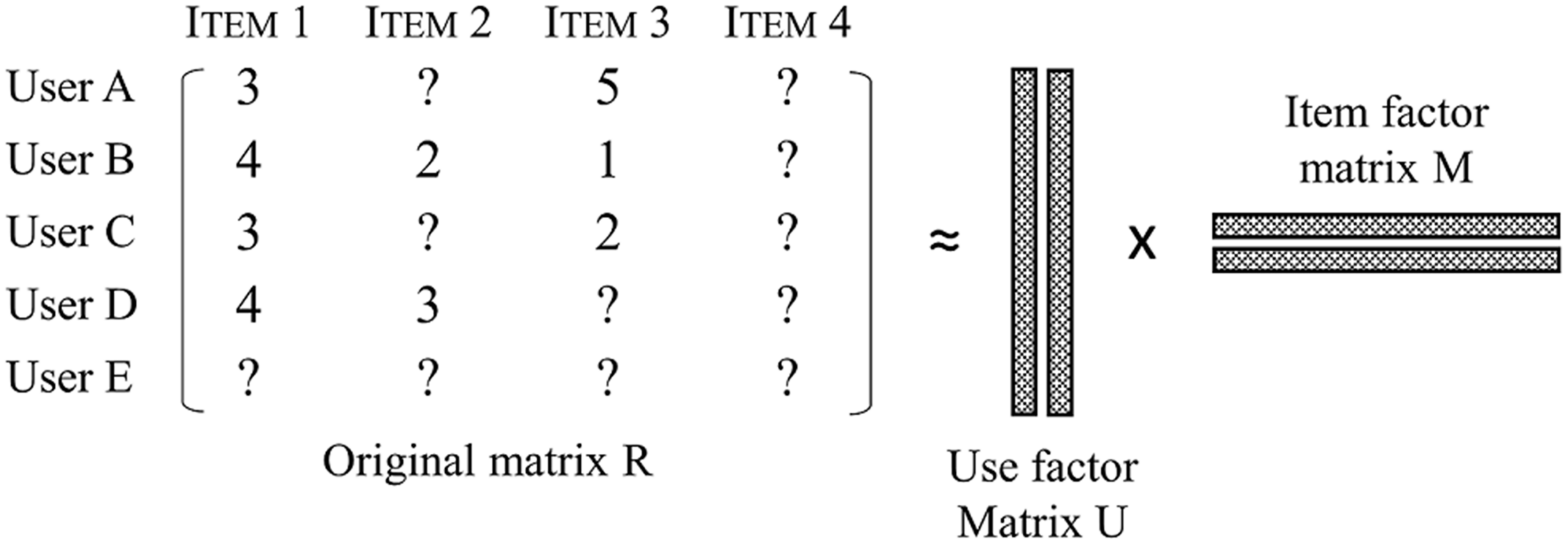
Matrix Factorization of user-item.

In collaborative filtering, a user-item matrix is used to predict the preference of the recommendation target customer, and a matrix value indicates the degree of satisfaction of the user with respect to the item. This is illustrated in Fig 3.

In Fig 3, ‘User A’ gave 3 and 5 rating values to ‘Item 1’ and ‘Item 3’, respectively. However, an evaluation value in the user-item matrix may either be for a new user whose data is concentrated according to the popularity of the product, or the evaluation value may not have been input yet. In such a case, it is impossible to predict the preference because there is not enough data to measure the similarity, which is known as a ‘*cold start*’. Also, if there is no score as in the ‘Item 4’ column, indicating it has not been purchased yet, it cannot be recommended until someone provides a score. Problems caused by the lack of data are collectively referred to as data sparseness. Various studies have been conducted to solve these problems [22–24]. As noted in a previous study, there is a method that uses keywords of the article instead of the using information of the article in order to recommend new articles which are lacking evaluation information or utilization information to users in the article recommendation system. When a new article is obtained, the keywords of the new article are compared with the keywords of the article selected by the user; then the probability that this article is recommended to each user is calculated, and a new article is recommended to the N users who are most likely to be recommended. The algorithm used here is the Naive Bayes model, which is often used in document classification [25]. In addition, it is difficult to provide a recommendation list for new customers such as ‘User E’ in Fig 3 because there is no purchase history information to understand the user’s taste. In order to solve this problem, an analysis that combines the collaborative filtering technique and the centrality of the social network technique has been attempted. First, the network is created based on the similarity between existing customers. Then, the highly centrally located users are determined as the neighbors of the new customers, and the selected items are recommended to the new customers. In this way, the network-based technique improves the recommendation accuracy by utilizing the information for all users with different buying tendencies, unlike the existing collaborative filtering technique [26].

In order to solve the problem of data scarcity, a method that reduces the dimension of the data has been studied. Typically, this involves singular value decomposition (SVD), which removes an insignificant user or item directly from the user- item matrix, thus reducing the dimension of the matrix [27, 28].

#### • Memory-based collaborative filtering

Memory-based collaborative filtering techniques can be divided into user-based collaborative filtering and item-based collaborative filtering. User-based collaborative filtering is a method that selects neighboring users who have a similar tendency to other customers by using user preference information, and then recommends common favorites to the selected neighbors [29]. Item-based cooperative filtering is a method in which a specific item is used as a criterion, a similar item ranked by a user is selected as a neighbor item, and predict the preference of the target user for the specific item [30].

#### • Model-based collaboration filtering

Memory-based collaborative filtering is considered as ‘Lazy Learning’, meaning that it derives the results via a heuristic technique whenever a recommendation is required without building a model. This has the advantage that the number of parameters that the user has to set is small, but there are also various problems such as data sparsity. Since this problem becomes more serious when applied to real data, much research has been conducted on this issue. In the present study, the benefits of model-based cooperative filtering, which recommends items after utilizing learning data, have been verified. It is possible to supplement the problems of memory-based collaborative filtering by applying machine learning and data mining techniques to both the similarity measurement and the preference prediction of existing memory-based collaborative filtering [31]. Recently, studies have been carried out to increase the accuracy of predicting preferences by applying appropriate models to detect complex patterns inherent in the data; such techniques have been recognized as being superior in performance when applied to actual data. In general, a classification model is used for data that indicates user preference as a categorization, and a regression model or a singular value decomposition technique is used for continuous data [32].

## Proposed system architecture

**Fig 4:**
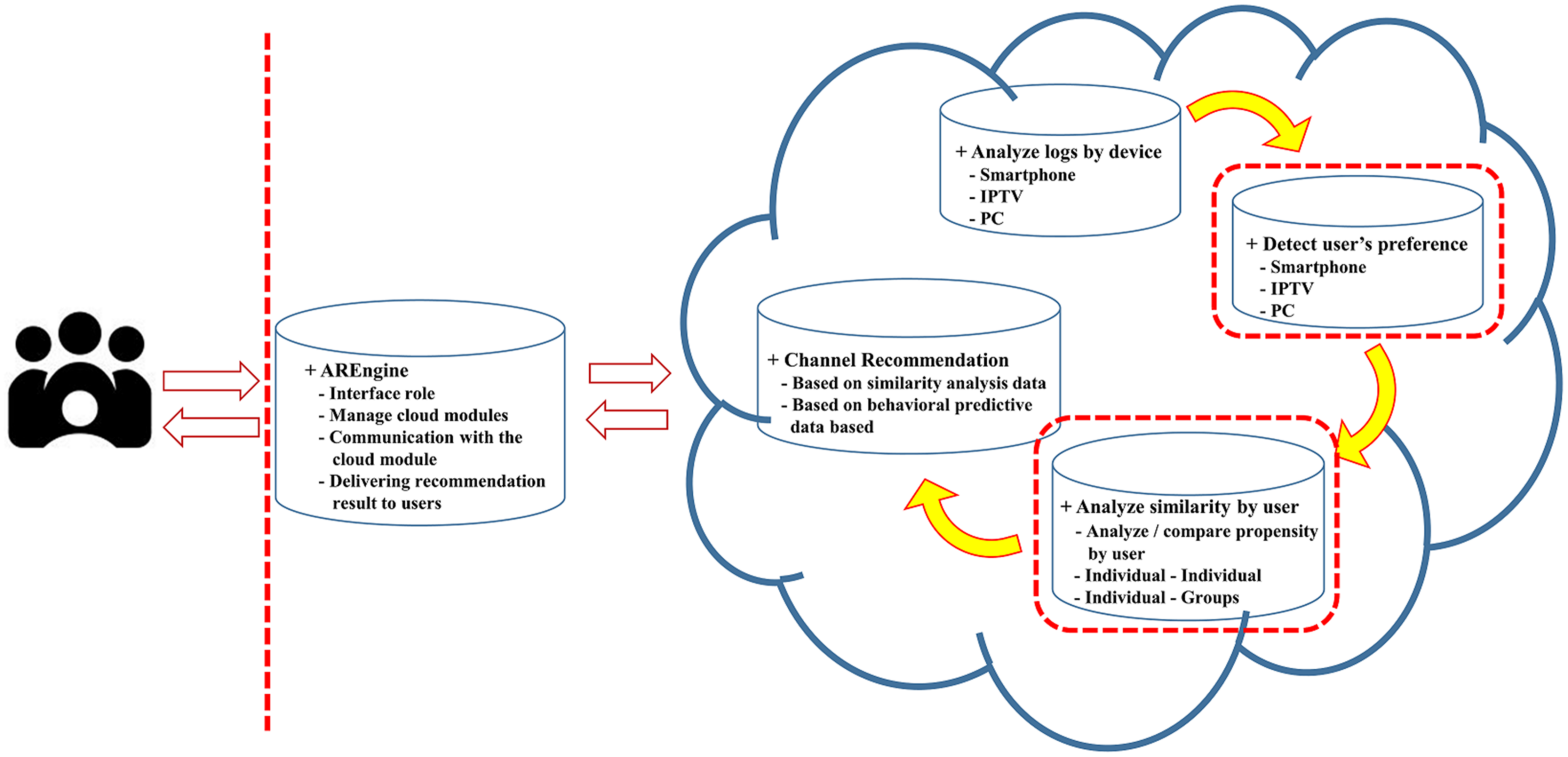
Proposed system architecture.

This section describes the proposed system architecture. The proposed system is shown in Fig 4.

### A. Architecture module configuration

The system consists of four modules that perform log analysis, preference detection, similarity analysis, and channel recommendation functions, each fulfilling different roles.

The **log analysis module** analyzes logs by device. Through this module, it is possible to analyze the log information of various devices such as smartphones, IPTVs, and PCs in an integrated manner.

The **preference detection module** detects user preference. This module analyzes the tendency of users based on the log information and analyzes information dealing with how much the channels and programs are preferred. For the analysis and clustering of a user’s watching pattern, the preference is analyzed based on the watching frequency, and the formula is as follows. Table 1 summarizes the mathematical notations used to explain the proposed recommendation system.

**Table 1:** The notations used in proposed system.

Notations	Description
$x_{p}^{u}$	The user ***u*** ’s preference for the program ***p***.
***N***_***p***_	The total number of broadcasts
$N_{p}^{u}$	The number of watching times
$N_{p^{\prime }}^{u}$	The preference of the user for a particular time series program ***p*′**
***L***_***p***′_	The total time of the program ***p*′**
***W***^***up***′^	The user ***u*** actually watched time at the program ***p*′** in the total time *L*_*p*′_
$P_{p^{\prime }}^{u}$	The user ***u*** preference for a specific program ***p*′**
$W_{p^{\prime }}^{u}$	The weekly statistical data $W_{p^{\prime }}^{u}$ for user preference for the program can be obtained by summing over all watched programs ***N***_***p***_.
$pw_{p^{\prime }}^{u}$	The reward value for a specific program ***p*′**
***FL***_***p***′_	The total length (time) of a specific program ***p*′**
***L***^***up***′^	The time when user ***u*** actually watched a specific program ***p*′**
$T_{p}^{u}$	The user ***u*** preference for the program
$R_{p}^{u}$	Real-time programs
$V_{p}^{u}$	VOD programs
$x_{p_{s1}}^{u}$	The user ***u***’s preference for *Section Video Clip* ***s1*** of program ***p***
$C_{p}^{u}$	The preference of user ***u*** for program ***p***
$p_{avg}^{u}$	The average for the preference for each interval

$$x_{p}^{u}=\frac{N_{p}^{u}}{N_{p}^{u}}$$ (1)

$$N_{p}^{u}=\sum_{p^{\prime }=1}^{N_{p}}N_{p^{\prime }}^{u}$$ (2)

$$N_{p^{\prime }}^{u}=\{\begin{array}{l} 1,if\frac{W^{up^{\prime }}}{L_{p^{\prime }}}>0.1\\ 0,otherwise \end{array}$$ (3)

$$x_{g}^{y}=\frac{\sum_{p\in g}x_{p}^{u}}{\sum_{g^{\prime }=1}^{N_{G}}x_{g^{\prime }}^{u}}$$ (4)

$$x_{c}^{u}=\frac{\sum_{p\in c}x_{p}^{u}}{\sum_{c^{\prime }=1}^{N_{C}}x_{c^{\prime }}^{u}}$$ (5)

Eq (1) represents the user ***u***'s preference $x_{p}^{u}$ for the program ***p***. The total number of broadcasts ***N***_***p***_ is divided by the number of watching times $N_{p}^{u}$. Eqs (2)–(5) show specific calculation methods for (1). In (2), $N_{p^{\prime }}^{u}$ is the preference of the user for a particular time series program ***p*′**. In (3), the time at which the user ***u*** actually watched the program ***p*′** in the total time ***L***_***p***′_ of the program ***p*′** is denoted ***W***^***up***′^ and $N_{p^{\prime }}^{u}$ is 1 if the actual watching time exceeds 10 percent of the total time, 0 otherwise. The preference can be obtained via the sum of these reward values for all watched programs.

The **similarity analysis module** analyzes the degree of similarity for each user. Based on the user’s channel and program preference information, this module analyzes whether there is a user who has a similar tendency among each user.

The **channel recommendation module** recommends the channel to the user. This module recommends channels and programs to users with similar tendencies based on the analyzed channels and program preferences and similarity information between users.

In the proposed system, a program is recommended using two pieces of information. When the preference is detected, the real-time preference of the broadcast and the preference of each broadcast section are separately analyzed to store the data. Then, each user is set as a vector, and the similarity between users is obtained by using the preference data. Programs and channels are then recommended based on the similarity.

In the proposed system, the user’s program preference is obtained as follows.

$$p_{p^{\prime }}^{u}=\sum_{p^{\prime }=1}^{N_{p}}w_{p^{\prime }}^{u}$$ (6)

$$w_{p^{\prime }}^{u}=\sum_{w_{i}}^{w_{n}}pw_{p^{\prime }}^{u},i=1;n=7$$ (7)

$$pw_{p^{\prime }}^{u}=\sum_{i=1}^{n}pw_{ip^{\prime }}^{u},n=3$$ (8)

$$pw_{1p^{\prime }}^{u}=\{\begin{array}{l} 1,if\frac{L^{up^{\prime }}}{FL_{p^{\prime }}}>0.1\\ 0,otherwise \end{array}$$ (9)

$$pw_{2p^{\prime }}^{u}=\{\begin{array}{l} 1,if\frac{L^{up^{\prime }}}{FL_{p^{\prime }}}>0.2\\ 0,otherwise \end{array}$$ (10)

$$pw_{3p^{\prime }}^{u}=\{\begin{array}{l} 1,if\frac{L^{up^{\prime }}}{FL_{p^{\prime }}}>0.3\\ 0,otherwise \end{array}$$ (11)

In Eq (6), $P_{p^{\prime }}^{u}$ calculates the user ***u*** preference for a specific program ***p*′** in the proposed system. The weekly statistical data $W_{p^{\prime }}^{u}$ for user preference for the program can be obtained by summing over all watched programs ***N***_***p***_. Eq (7) is a method for obtaining the program preference for one week, where $pw_{p^{\prime }}^{u}$ is the reward value for a specific program ***p*′**. We can confirm that the total program preference is for one week. Eq (8) shows how to obtain the program preference. In the proposed system, to increase the accuracy of the recommendation, the watching time is considered to be 10–30% of the program video length. Eqs (9)–(11) indicate that the reward value is stored separately when the actual watching time is 10%, 20%, and 30%. The program’s preference can be obtained from the sum of these three types of data. In here, ***FL***_***p***′_ represents the total length (time) of a specific program ***p*′. *L***^***up***′^ is the time when user ***u*** actually watched a specific program ***p*′**. The separated data can be divided into real-time programs and VOD programs.

$$T_{p}^{u}=R_{p}^{u}+V_{p}^{u}$$ (12)

$$R_{p}^{u}=W_{p}^{u}\cdot wt$$ (13)

$$V_{p}^{u}={W^{\prime }}_{p}^{u}\cdot wt$$ (14)

$$T_{p}^{u_{i}}=\left(\left(\sum_{w_{i=1}}^{w_{n=7}}\left(\sum_{i=1}^{n=3}pw_{ip^{\prime }}^{u}\right)\right)\cdot wt\right)+\left(\left(\sum_{w_{i=1}}^{w_{n=7}}\left(\sum_{i=1}^{n=3}p{w^{\prime }}_{ip^{\prime }}^{u}\right)\right)\cdot wt\right)$$ (15)

Eq (12) shows that the user ***u*** preference $T_{p}^{u}$ for the program is divided and processed into real-time programs $R_{p}^{u}$ and VOD programs $V_{p}^{u}$. The preferences for real-time programs $R_{p}^{u}$ and VOD programs $V_{p}^{u}$ can be obtained by (13) and (14), where ***wt*** is weight value.

Eq (15) shows how to calculate the preference in consideration of real-time programs and VOD programs using Eq (12). Using Eqs (9)–(11), we calculate the preference of the program ***p*′** according to the length of the watching time for each weekday for a week. In the same way, the preference for VOD programs can be calculated. If we want to give weight to specific programs, we can obtain program preference by assigning weight value ***wt***.

The similarity of two users is measured based on the preference value of the real-time program and the VOD program obtained by the above equation. At this time, the cosine similarity measure is used to determine the similarity between vectors. The cosine similarity is given as follows.

$$cos\left(\theta \right)=\frac{A\cdot B}{\left\|A\right\|B\|}=\frac{\sum_{i=1}^{n}A_{i}\cdot B_{i}}{\sqrt{\sum_{i=1}^{n}{\left(A_{i}\right)}^{2}\cdot }\sqrt{\sum_{i=1}^{n}{\left(B_{i}\right)}^{2}}}$$ (16)

$$sim\left(u_{i},u_{j}\right)=\frac{\sum_{i=1}^{n}T_{p}^{u_{i}}\cdot T_{p}^{u_{j}}}{\sqrt{\sum_{i=1}^{n}{\left(T_{p}^{u_{i}}\right)}^{2}\cdot }\sqrt{\sum_{i=1}^{n}{\left(T_{p}^{u_{j}}\right)}^{2}}}$$ (17)

### B. Channel scheduling system

**Fig 5:**
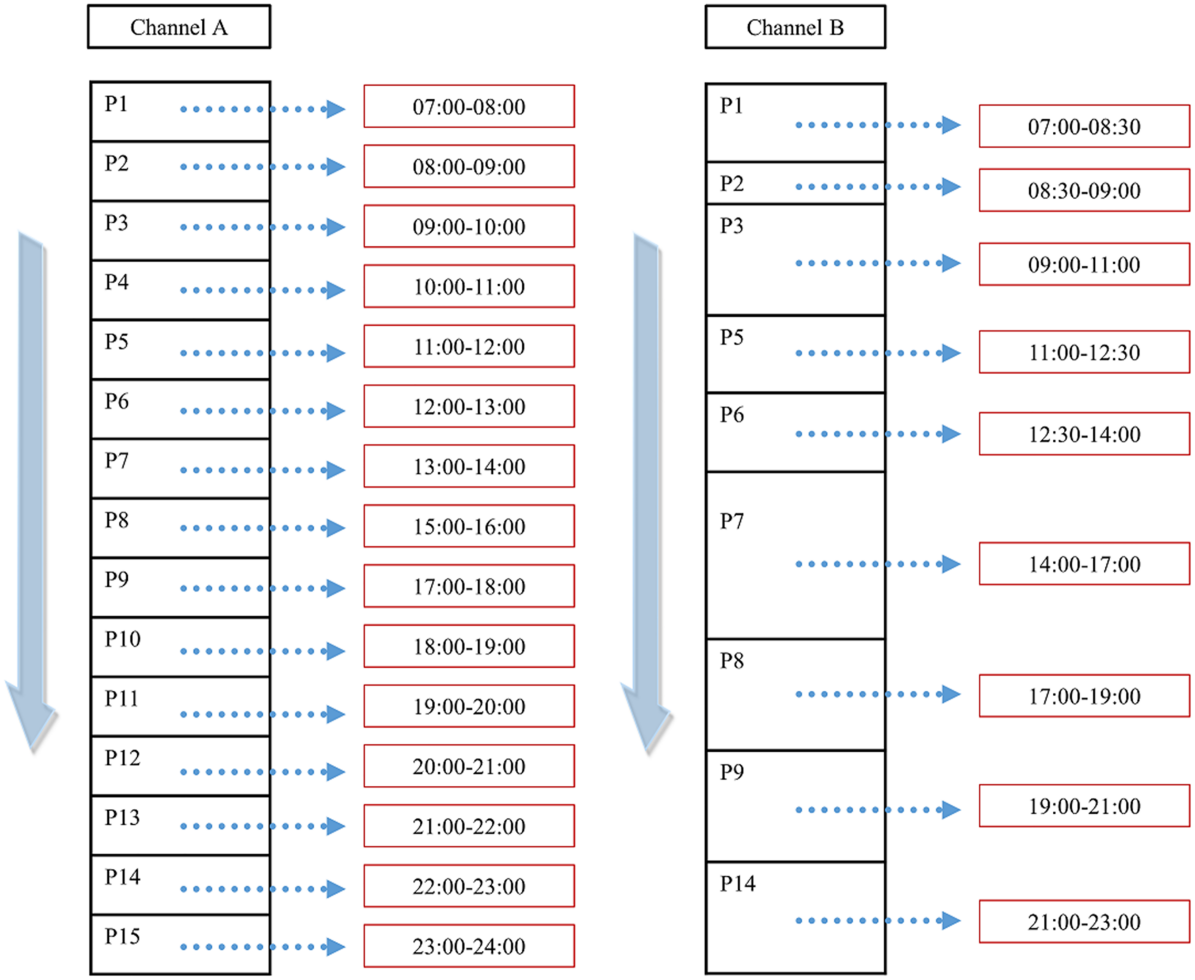
Conventional channel and program classification.

The following describes the channel scheduling system proposed in this paper. In the conventional channel and program classification methods, broadcast contents are broadcasted at predetermined time intervals for each channel so that the user can watch the broadcast only by accessing the corresponding channel at a predetermined time. This is illustrated in Fig 5.

**Fig 6:**
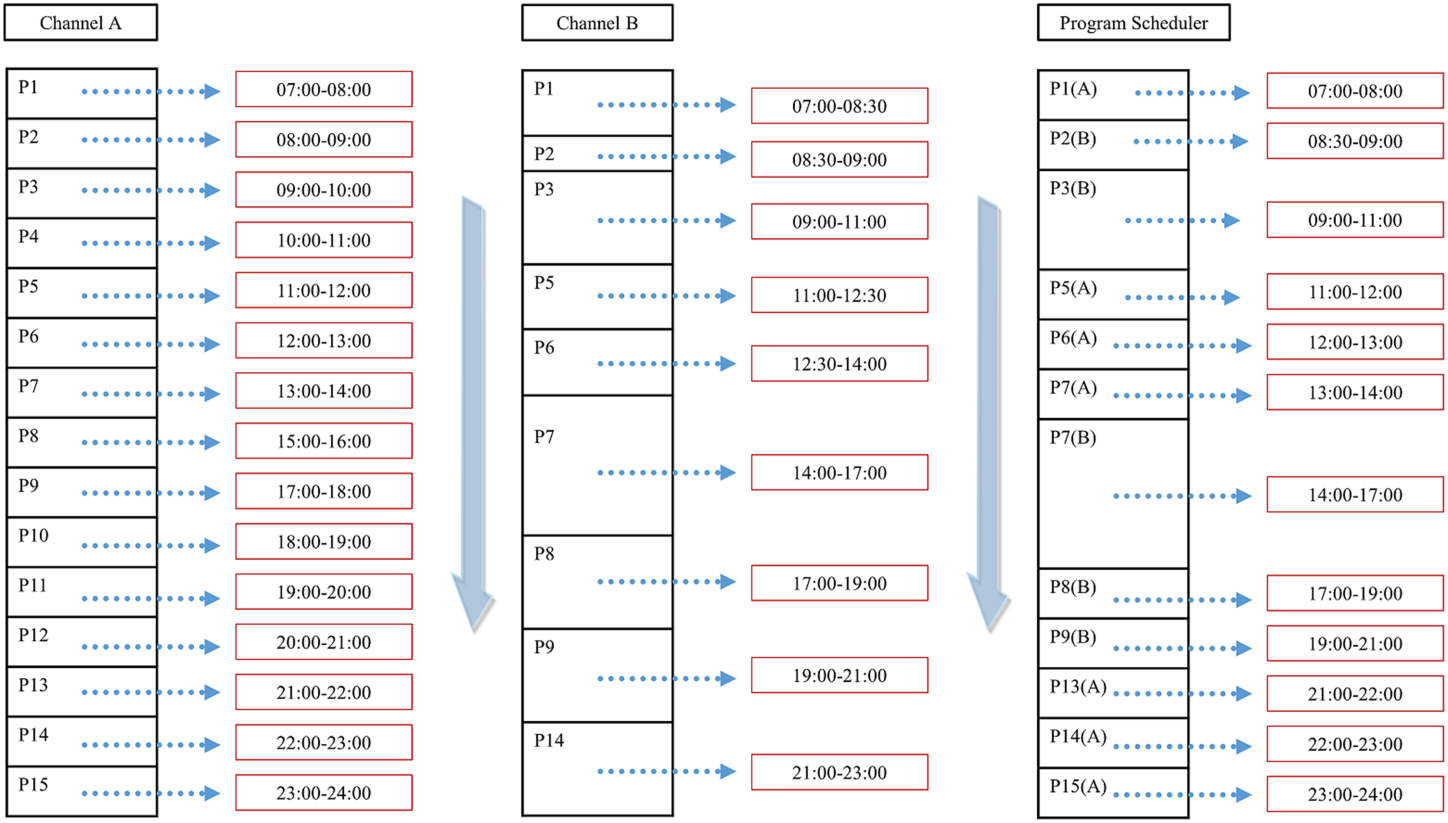
Individual scheduling of the programs.

In the current TV system, programs are classified according to channels. Therefore, it is impossible to schedule by program. On the other hand, in this paper, we propose a recommendation method for each program and a method for individual scheduling of the program based on this recommendation method. A schematic of the idea is shown in Fig 6.

Since the system constituting the contents can be classified according to the program, the proposed method can store a program desired by the user, and thus create and schedule a kind of individual channel.

**Fig 7:**
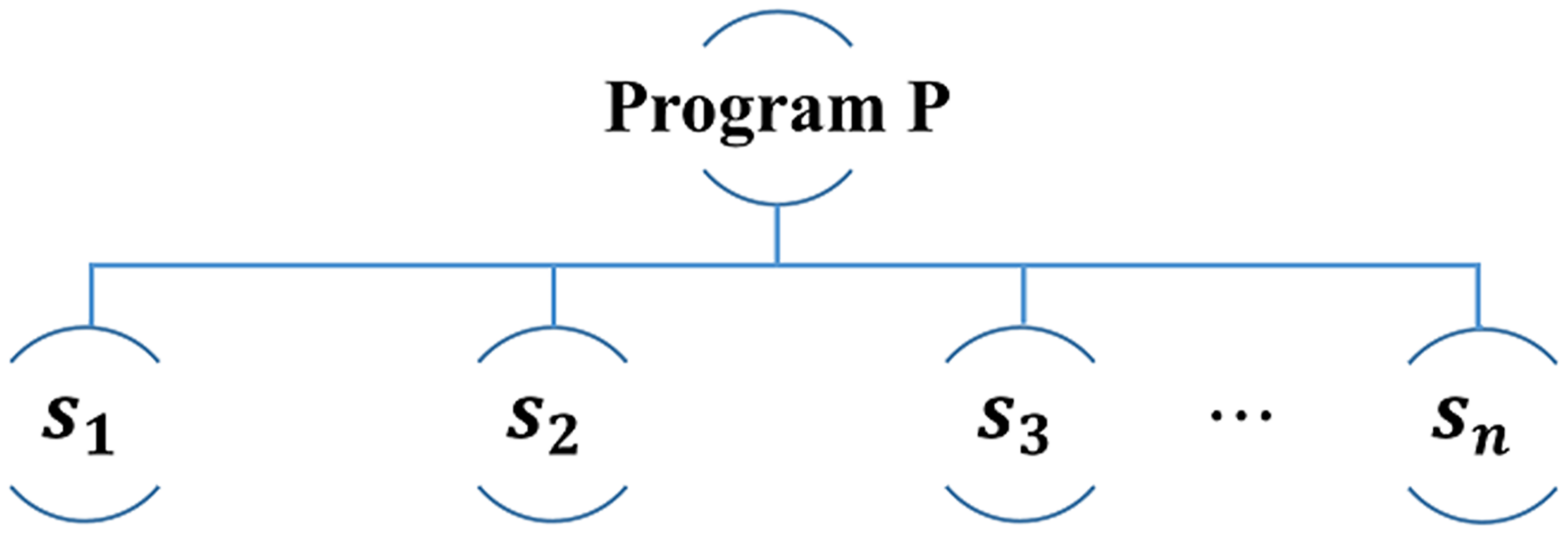
Section video clips of program *p*.

In this paper, we recommend a program to users by considering both the preference for the *Full Video Clip* of the program and the preference for the *Section Video Clip* of the program. Program ***p*** consists of several *Section Video Clips* as shown in Fig 7.

The *Section Video Clip* is represented by the notation $x_{p_{s1}}^{u}$. $x_{p_{s1}}^{u}$ is the user ***u***'s preference for *Section Video Clip* ***s1*** of program ***p***. If a program is composed of four *Section Video Clips*, the preference for the corresponding *Section Video Clips* can be obtained differently. Therefore, the program can have a preference value based on watching time and a preference value for each *Section Video Clip*. At this time, since several preference values are generated for the *Section Video Clip*, an average value needs to be selected to represent each clip.

In this paper, we use the average value of all preference values of *Section Video Clips* as a representative value of the *Section Video Clip* preference value, which is expressed by (18).

$$p_{avg}^{u}=(x_{p_{s1}}^{u}+\cdots +x_{p_{sn}}^{u})/n$$ (18)

The similarity of *Section Video Clip*s can be obtained in the same way as in (15). Finally, the preference for program recommendation can be obtained as in (19). $C_{p}^{u}$ is the preference of user ***u*** for program ***p***, and can be expressed as the sum of user ***u***'s preference $x_{p}^{u}$ for the program ***p*** and average $p_{avg}^{u}$ for the preference for each interval.

$$C_{p}^{u}=x_{p}^{u}+p_{avg}^{u}$$ (19)

If the program is recommended in this way, it is possible to obtain the preference and similarity for each program. Therefore, it is possible to recommend only a program that cannot be used in the existing channel recommendation method. At this time, since the preference for the *Section Video Clip* is reflected as the representative value for the calculation, the reliability of the prediction can be increased when a program is recommended.

### C. Preference prediction performance by weights

The prediction accuracy depends on the weight value that is used when calculating the program preference. We analyzed the difference in degree of prediction for the weighting methods of both the proposed method and the existing weighting method.

The existing method is denoted by W1 and the method proposed in this paper is denoted by W1-3. We tested 10, 20, 30, and 40 of the user’s top preference programs using the two methods. The higher the result, the better the performance.

When calculating the number of interested watching times, the existing method includes the program in the number of watched times when the actual watching time of the program is more than 10% of the watching time. However, the proposed method reflects the user’s interest more precisely by reflecting the actual watching time between 10% and 30%.

Table 2 shows that the proposed method positively affects the rank ordering of user data because it uses variable weights instead of fixed weights.

**Table 2:** Preference with different weight values.

Method	P10	P20	P30	P40
W 1	0.611	0.593	0.572	0.551
W 1–3	0.725	0.682	0.642	0.601

## Experiment and evaluation

In this section, in order to evaluate the validity of the proposed idea, we compared the proposed method against the algorithm used in existing channel recommendation methods.

We used a set of real TV watching history data which consists of the watched TV programs by 3,318 TV users for 6 months from Jan. 1, 2015 to June 30, 2015 by a TV watching rate survey agency, Total National Multimedia Statistics (TNMS) Research [33]. Currently, this data is used for commercial purposes and needs to be purchased for use. The experimental method is as follows.

We compared the prediction accuracy of the proposed algorithm with that of the existing algorithms by using the audience record of each viewer as the training data set. The prediction accuracy is expressed by (20).

$$Precision=\frac{tp}{tp+fp}$$ (20)

The precision is the percentage of recommended TV programs that have been recommended, and the closer this value is to 1, the higher the accuracy. ***tp*** is the number of programs that actually succeeded in recommendation, and ***fp*** is the number of failed recommendations.

We used the user-based collaborative filter (UBCF), item-based collaborative filter (IBCF) and RAMDOM (random) method for the evaluation. The results of the experiment are shown in Table 3.

**Table 3:** Program precision.

Method	Precision (%)
UBCF	32.12
IBCF	34.25
Random	29.23
Proposed approach	39.61

Experimental results exhibit 32.12%, 34.25%, and 29.23% for the prediction accuracy for the UBCF, IBCF, and Random methods, respectively. On the other hand, when the proposed method is used, the result is 39.61%, demonstrating a higher accuracy.

## Conclusions

In this paper, we propose a collaborative filtering TV program recommendation system that uses the watching time of each channel and the watching time of each *Section Video Clip* for the preference calculation. To this end, the watching rate is diversified and reflected in the calculation, as well as the watching rate of the *Section Video Clip*. And, since the system constituting the contents can be classified according to the program, the proposed method can store a program desired by the user, and thus create and schedule a kind of individual channel. Experimental results show that the proposed method has a higher prediction accuracy than existing algorithms in channel preference. Based on this research, we will study the efficiency of the channel scheduling system in future studies.
